# Expression of 5-lipoxygenase and 15-lipoxygenase in rheumatoid arthritis synovium and effects of intraarticular glucocorticoids

**DOI:** 10.1186/ar2717

**Published:** 2009-06-04

**Authors:** Karina Roxana Gheorghe, Marina Korotkova, Anca Irinel Catrina, Linda Backman, Erik af Klint, Hans-Erik Claesson, Olof Rådmark, Per-Johan Jakobsson

**Affiliations:** 1 Department of Biosciences and Nutrition, Novum, Karolinska Institute, SE-141 57 Huddinge, Sweden; 2 Department of Medicine, Rheumatology Unit, Karolinska University Hospital and Karolinska Institute, S-171 76 Stockholm, Sweden; 3 Orexo AB, Virdings allé 32 A, SE-751 05 Uppsala, Sweden; 4 Department of Medical Biochemistry and Biophysics, Karolinska Institutet, SE-171 77 Stockholm, Sweden; 5 Karolinska Biomic Center, Karolinska University Hospital and Karolinska Institute, S-171 76 Stockholm, Sweden

## Abstract

**Introduction:**

It was previously shown that lipoxygenase (LO) pathways are important in the rheumatoid arthritis (RA) inflammatory process and that synovial fluid from RA patients contains high amounts of leukotrienes. We therefore aimed to investigate the 5-LO and 15-LO-1 expression pattern in RA and ostheoarthritis (OA) synovial tissue and to study the effect of intraarticular glucocorticoid (GC) therapy on enzyme expression.

**Methods:**

Expression of LOs was evaluated by immunohistochemistry in RA and OA synovial biopsies. Cellular localization of these enzymes was analyzed by double immunofluorescence. In synovial biopsies from 11 RA patients, 5-LO and 15-LO-1 expression was evaluated before and after triamcinolone hexacetonide knee injection and assessed by image analysis to quantify their expression. We also investigated the presence of 15-LO-1 by immunohistochemistry in synovial fluid (SF) cells as well as their ability to form 15-hydroxyeicosatetraenoic acid (15-HETE) following treatment with arachidonic acid (AA).

**Results:**

5-LO and 15-LO-1 are present in RA and OA synovium, with 5-LO being mostly expressed in lining and sublining macrophages, neutrophils and mast cells and 15-LO-1 mainly in lining macrophages, fibroblasts and sublining endothelial cells. Intraarticular GC treatment resulted in a significant suppression of 5-LO expression, but did not influence the 15-LO-1 enzyme significantly. Also, SF cells express a functional 15-LO-1 and produce 15-HETE when challenged with AA.

**Conclusions:**

These data demonstrate that local therapy with GC decreases 5-LO expression in RA synovium and offer an additional possible mechanism for the efficiency of intraarticular adjuvant therapy in RA.

## Introduction

Rheumatoid arthritis (RA) is a chronic inflammatory disease characterized by polyarticular joint inflammation, synovial hyperplasia, and cartilage and bone destruction, with subsequent joint deformities. The inflammatory synovial fluid in RA patients contains–in addition to various cytokines and growth factors–high levels of leukotrienes, with leukotriene B_4 _(LTB_4_) being predominant [1].

LTB_4 _is a powerful proinflammatory lipid mediator and one of the most potent chemotactic agents known to date [2]. This leukotriene is produced mainly by neutrophils, macrophages and mast cells, and promotes neutrophil recruitment and activation [3]. Neutrophils are the most abundant leukocytes in rheumatoid joints [4], and have destructive potential by secreting proteases and reactive oxygen species and by promoting synthesis of matrix metalloproteinases [5,6]. Several lines of evidence have implicated LTB_4 _as an important mediator of joint inflammation in RA. LTB_4 _is present at higher levels in serum of patients with active RA compared with patients with inactive arthritis or normal subjects [7], and its levels correlate with the disease severity [8].

A critical contribution of neutrophil-derived LTB_4 _to arthritis induction and severity has recently been revealed in a mouse serum transfer model of inflammatory arthritis [9]. In this study it was shown that mice lacking 5-lipoxygenase (5-LO) or leukotriene A_4 _hydrolase enzymes are protected from developing the disease and that there is a specific requirement for LTB_4 _and not other leukotrienes for the pathogenesis in this model. 5-LO and 5-LO activating protein (FLAP), followed by leukotriene A_4 _hydrolase, are the enzymes responsible for the sequential formation of LTB_4 _from arachidonic acid (AA).

15-Lipoxygenase (15-LO) is a lipid-peroxidizing enzyme mainly expressed in airway epithelial cells, eosinophils, reticulocytes and macrophages. In humans, 15-LO exists as two different enzymes with different cell localizations and product profiles [10]. 15-LO-1 converts AA to an unstable intermediate, 15-hydroperoxyeicosatetraenoic acid, which can be further converted to 15-hydroxyeicosatetraenoic acid (15-HETE). The 15-LO-1 enzyme has proinflammatory actions, with high levels of 15-HETE reported in sputum of asthmatic patients along with increased macrophage 15-LO-1 mRNA expression [11]. 15-LO-1 expression is induced by IL-13 in human blood monocytes [12] and by IL-4 in monocytes, alveolar macrophages, dendritic cells, mast cells and rheumatoid arthritis synovial cells [12-18]. Only recently was it reported that 15-LO-1 can catalyze the metabolism of AA to the proinflammatory eoxins that can increase permeability of the endothelial cell monolayer *in vitro*, indicating that they can enhance vascular permeability [19]. 15-LO-1 products, however, were also demonstrated to have protective roles in inflammatory disorders due to formation of anti-inflammatory lipoxins [20-22]. The 15-LO-1 mRNA was demonstrated to be present in RA synovial membranes [23] and its expression was stronger in RA compared with osteoarthritis (OA) biopsies [24].

The 5-LO cascade and the role of LTB_4 _in RA are well documented. Although the presence of 5-LO enzyme in the synovial lining of rheumatoid tissue has recently been reported [24], a detailed characterization of cells expressing 5-LO in human synovial tissue is lacking. Evidence is also limited regarding the influence of current therapy for RA on this pathway.

Glucocorticoids (GCs) are used in RA as an efficient adjuvant therapy and their efficacy is related to their broad anti-inflammatory profile, with inhibition of inflammatory cells functions [25]. Controversial results have been reported about the effects of GCs on 5-LO expression and LTB_4 _formation. Some studies reported that 5-LO pathway activity is decreased in the presence of GCs [26,27], while other investigators have shown that *in vivo *GC administration had no influence on LTB_4 _formation [28,29]. In contrast, leukotriene synthesis and 5-LO expression were increased in human blood monocytes [30] and mast cells [31] by dexamethasone. In addition, blood polymorphonuclear neutrophils from RA patients released higher amounts of LTB_4 _after GC pulse therapy [32] while intraarticular corticosteroids reduced the LTB_4 _level in synovial fluid of RA patients [33].

In comparison, there are few studies to date investigating the effects of corticosteroids on 15-LO-1 expression. In a rabbit model for atherosclerosis, corticosteroid treatment was shown to decrease atherosclerotic plaque formation along with increasing 15-LO-1 expression in the arterial wall [34]. GC treatment of asthma patients, however, has been reported to decrease the expression of 15-LO-1 in the lung [35].

In the present study we characterized the expression pattern of 5-LO and 15-LO-1 enzymes in synovial tissue of RA and OA patients and phenotyped the positive cells. In addition, we determined the effects of intraarticular glucocorticoids on the expression of these enzymes in RA synovium.

## Materials and methods

### Patients

In the first study group, we analyzed synovial biopsies from six RA patients and from five OA patients collected at the time of orthopedic surgery. In a second group, 11 RA patients were recruited into the study. The demographical and clinical data of the second patients group are presented in Table 1.

**Table 1:** Demographical and clinical data of the second patient group (n = 11)

Characteristic	Value
Age (years)	68 (35 to 83)
Gender (male/female)	3/8
Disease duration (months)	24 (3 to 240)
Current knee arthritis episode duration (months)	2 (0.5 to 6)
Taking disease-modifying antirheumatic drugs	6
Taking oral corticosteroids	2
Taking nonsteroidal anti-inflammatory drugs	4
Time between biopsies (days)	10 (7 to 12)

Data presented as median (range) or number of patients.

All patients in the second group received an intraarticular knee injection of 40 mg triamcinolone hexacetonide, and synovial biopsies were collected by arthroscopy immediately prior to treatment and a median of 10 days after treatment. The treatment regimen remained unchanged from at least 2 weeks prior to and during the entire study period.

All RA patients fulfilled the 1987 American College of Rheumatology diagnostic criteria for RA [36]. The ethics committee at the Karolinska Hospital approved all experiments on human cells and tissues. Informed consent was obtained from all study subjects.

### Tissue preparation and immunohistochemical analysis

Serial cryostat sections (7 μm) were fixed for 20 minutes in 2% formaldehyde (v/v), air-dried and then stored at -70°C. Immunohistochemical staining was performed as described previously [37]. The inhouse antibodies used were affinity-purified rabbit polyclonal antibody against human 5-LO and rabbit polyclonal anti-human 15-LO-1 antibody. Rabbit IgG served as the negative control. Stained synovial biopsies were evaluated using a Polyvar II microscope (Reichert-Jung, Vienna, Austria) and photographs were taken with a digital camera (300F; Leica, Cambridge, UK). Synovial expression of 5-LO and 15-LO-1 was quantified by computer-assisted image analysis and was expressed as the percentage of positive stained area versus total tissue area.

Synovial fluid cells from RA patients were collected on slides by cytospin centrifugation. The slides were then fixed and processed for immunhistochemical detection of 15-LO-1 as described above.

### Immunofluorescence staining

Double immunofluorescence staining was performed using rabbit anti-human 5-LO or 15-LO-1, mouse anti-human CD163 (Ber-MAC3; DakoCytomation, Glostrup, Denmark), mouse anti-human CD68 (KP1; DakoCytomation), mouse anti-human prolyl 4-hydrolase (DakoCytomation), mouse anti-human CD66b (80H3; Beckman Coulter, France), mouse anti-human CD3 (SK7; BD Biosciences, San Jose, CA, USA), mouse anti-human CD20 (DakoCytomation), mouse anti-human CD31 (EN4; Novakemi AB, Handen, Sweden), and mouse anti-human mast cell tryptase (Chemicon International, Temecula, CA, USA) antibodies.

The staining procedure has been published previously [38]. Briefly, after blocking with an avidin–biotin kit (Vector Laboratories, Peterborough, UK), sections were incubated overnight with primary antibodies. Subsequently, slides were incubated with secondary biotinylated goat anti-rabbit antibody (heavy and light chain; Vector Laboratories) and streptavidin-conjugated fluorochrome Alexa 488 (Molecular Probes, Leiden, the Netherlands). The slides were blocked again with the avidin–biotin kit and were incubated with the next secondary biotinylated horse anti-mouse antibody (IgG heavy and light chain; Vector Laboratories), followed by streptavidin-conjugated fluorochrome Alexa 546 (Molecular Probes). Matched IgG isotype controls were included for all markers.

### 15-LO-1 product measurement in RA synovial fluid cells

Synovial fluid from RA patients was centrifuged and the pelleted cells were resuspended in PBS and washed twice. The cellular composition of synovial fluid cells was analyzed using flow cytometry. Monocyte, neutrophil and lymphocyte populations were identified using a FACSCalibur (Becton Dickinson, San Jose, CA, USA) and Cell Quest software (Becton Dickinson). AA was added to a final concentration of 40 μM and the cells were incubated for 5 minutes at 37°C. Buffer control without cells was used to assess for spontaneous degradation of AA. Subsequently, the samples were centrifuged and the supernatant collected and stored at -70°C until analysis by enzyme immunoassay according to the manufacturer's instructions (Cayman Chemicals, Ann Arbor, MI, USA).

### Statistical analysis

Statistical analysis was performed using the Wilcoxon test and Bonferroni correction for multiple comparisons for paired samples for the synovial biopsy data, and using the Mann–Whitney test for 15-HETE production.

## Results

### RA synovial tissue displays a higher expression of 5-LO and 15-LO-1 enzymes compared with OA samples

**Figure 1:**
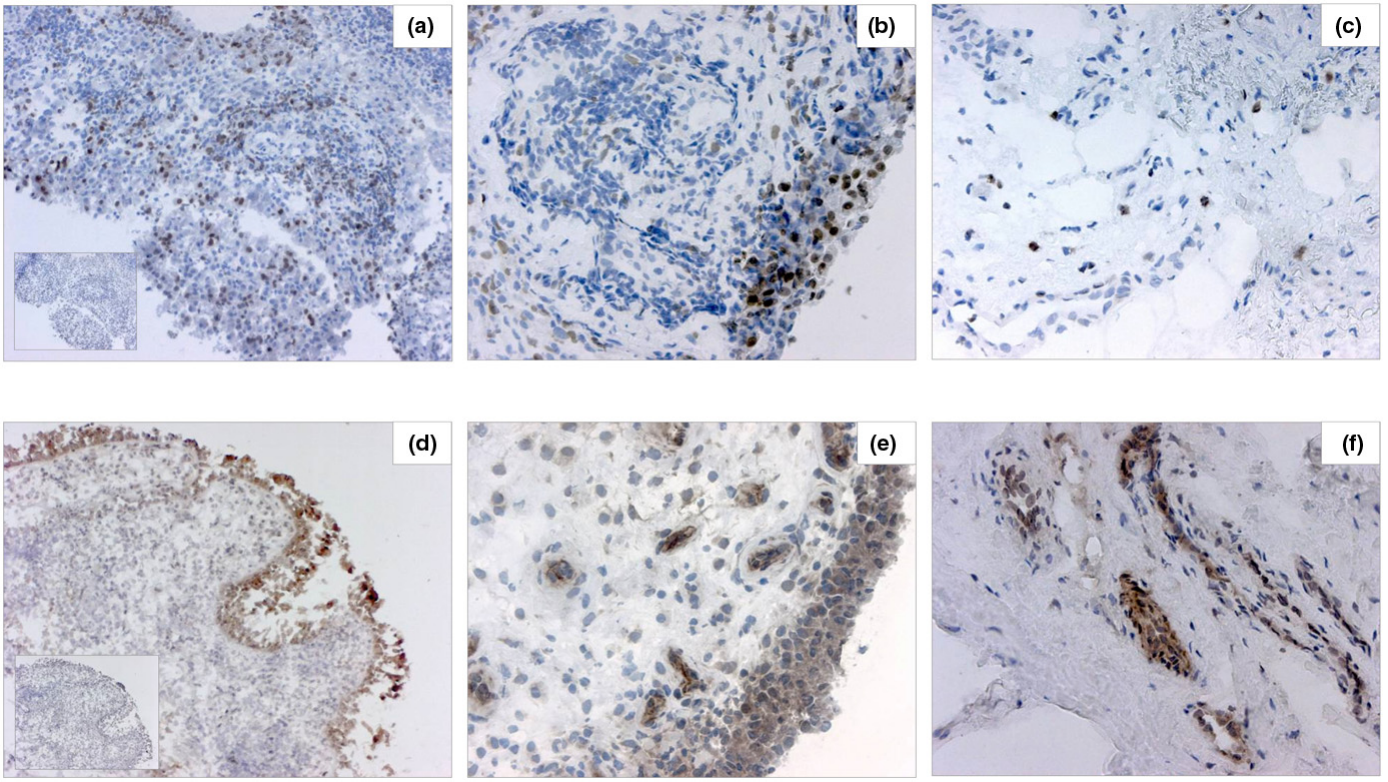
Lipoxygenase enzymes in rheumatoid arthritis and osteoarthritis synovial tissue. 5-Lipoxygenase (5-LO) and 15-LO-1 enzymes are present in both rheumatoid arthritis (RA) and osteoarthritis (OA) synovial tissue. Photographs illustrating brown (diaminobenzidine) immunoperoxidase staining for **(a, b, c) **5-LO and **(d, e, f) **15-LO-1 in sections from frozen synovial biopsies of (a, b, e) RA and (c, f) OA patients (hematoxylin counterstained). (d) Bronchial epithelium staining positive for 15-LO-1. Insets: (a) RA synovium stained with 5-LO antibody and (d) bronchial epithelium stained with 15-LO-1 antibody, preabsorbed with purified 5-LO and 15-LO-1 protein, respectively. Original magnification: ×100 (a, d and insets) and ×200 (b, c, e, f).

We detected intracellular 5-LO staining in all RA samples studied. Sections incubated with the preadsorbed 5-LO antibody showed no significant staining, confirming the specificity of the antibody for the 5-LO enzyme (Figure 1a, inset). Strong 5-LO staining was shown in macrophage-like cells within the synovial lining layer and in sublining tissue (Figure 1a,b). 5-LO positivity was scarce in the follicular mononuclear infiltrates, with the majority of patients not having detectable staining in these areas. By contrast, 15-LO-1 showed a very strong staining pattern mainly in the synovial lining cells and in vessels, with lower expression in scattered sublining macrophage-like and fibroblast-like cells (Figure 1e). The specificity of 15-LO-1 antibody was tested in bronchial tissue, and the airway epithelium was strongly stained (Figure 1d). In contrast, there was no staining after preincubation with the 15-LO-1-specific peptide against which the antibody was raised (Figure 1d, inset).

The OA synovial samples displayed mostly large areas of fibrosis and cartilage, with limited synovial membranes. Positive staining for 5-LO and 15-LO was detected almost exclusively in the synovial membrane areas. OA tissue showed low expression of both 5-LO and 15-LO-1 enzymes, with few stained cells scattered in the synovial membrane areas (Figure 1c,f). Strong staining for 15-LO-1, however, was detected in blood vessel cells.

**Figure 2:**
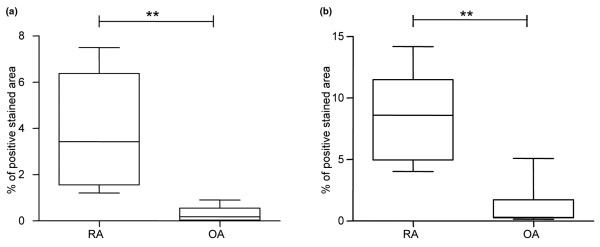
Osteoarthritis versus rheumatoid arthritis synovial expression of lipoxygenase enzymes. Ostheoarthritis (OA) synovial tissue displays a lower expression of 5-lipoxygenase (5-LO) and 15-LO-1 compared with rheumatoid arthritis (RA) synovium. Graphs show computer assisted-image analysis results for **(a) **5-LO and **(b) **15-LO-1 expression in RA tissue (n = 6) and OA tissue (n = 5). Results expressed as percentages of the total area of counterstained tissue. Horizontal lines, median values; whiskers, range values. **P < 0.01.

We then quantitatively analyzed the expression of LO enzymes in RA and OA synovial tissue sections. Both 5-LO and 15-LO-1 showed a lower expression in OA synovial tissue compared with RA samples (Figure 2).

### Phenotype of cells expressing 5-LO and 15-LO in RA synovium

**Figure 3:**
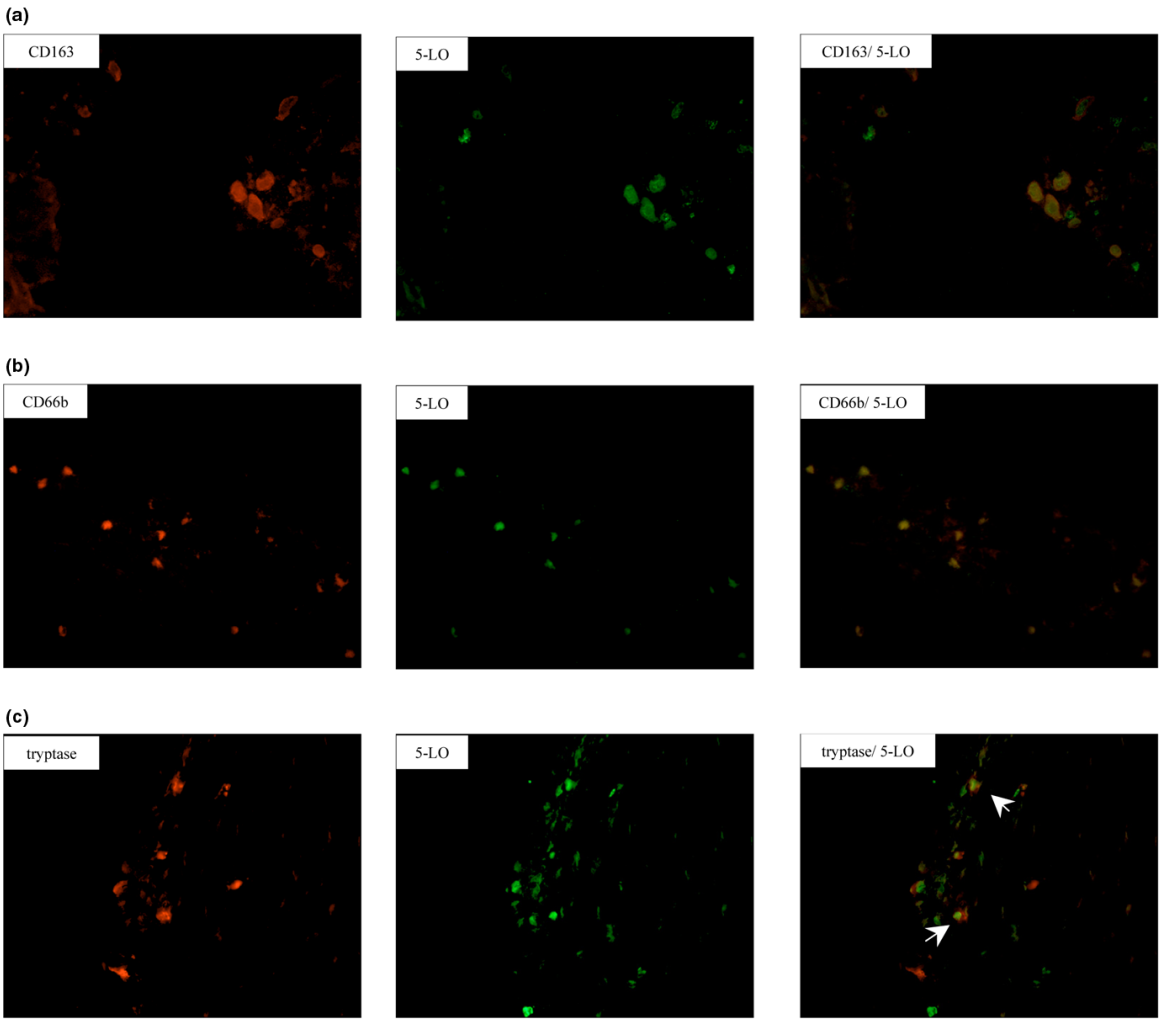
Synovial distribution pattern of 5-Lipoxygenase. CD163^+ ^macrophages, CD66b^+ ^neutrophils and tryptase-positive mast cells express 5-lipoxygenase (5-LO) in rheumatoid arthritis synovium. Photomicrographs showing fluorescent staining of **(a) **CD163^+ ^cells, **(b) **CD66b^+ ^cells and **(c) **tryptase-positive cells (Alexa 546, red), 5-LO-positive cells (Alexa 488, green), and superimposed staining. White arrows, double-positive mast cells expressing 5-LO. Original magnification: ×400.

**Figure 4:**
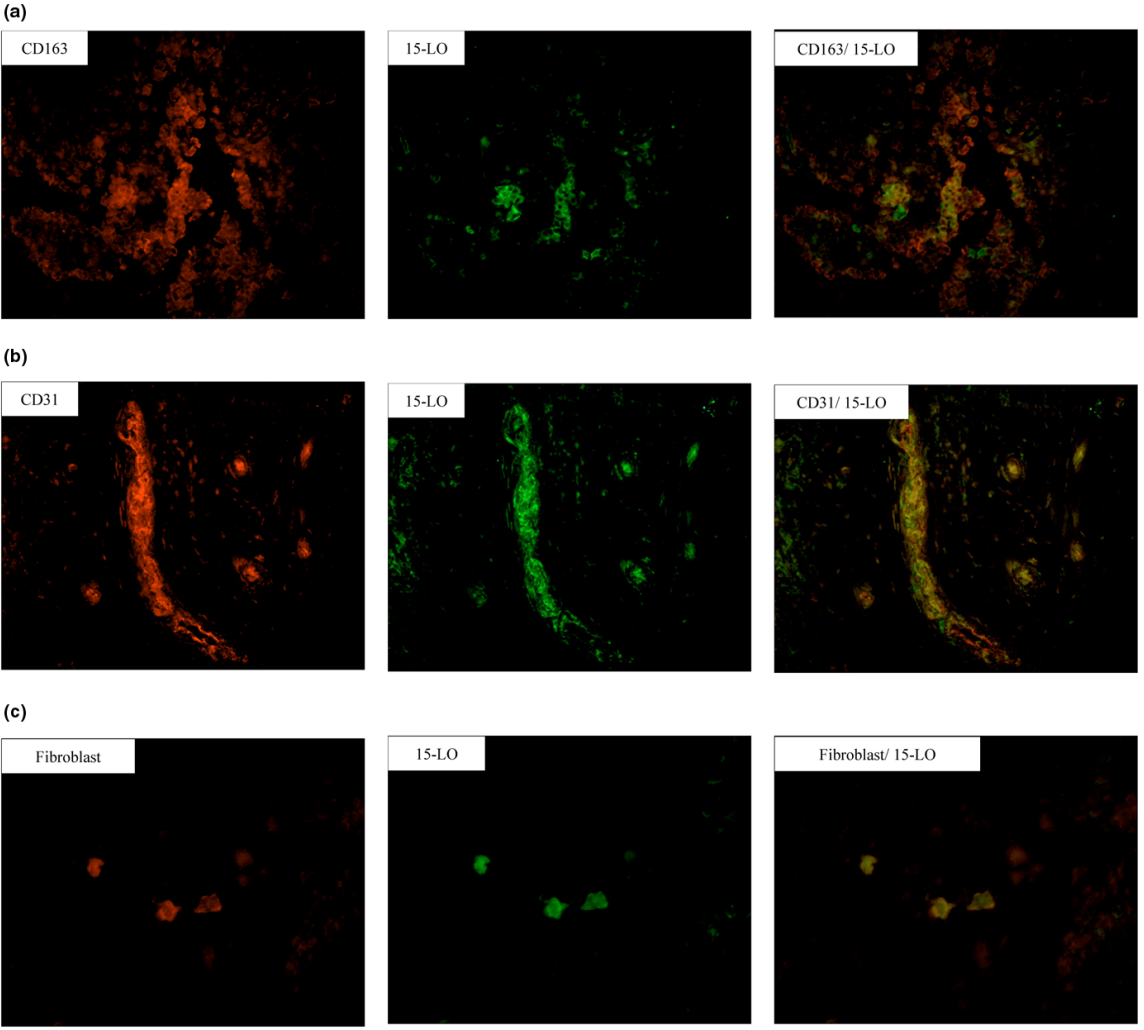
Synovial distribution pattern of 15-Lipoxygenase-1. CD163^+ ^macrophages, CD31^+ ^endothelial cells and prolyl 4-hydrolase-positive fibroblast cells express 15-lipoxygenase-1 (15-LO-1) in rheumatoid arthritis synovium. Photomicrographs showing fluorescent staining of **(a) **CD163^+ ^cells, **(b) **CD31^+ ^cells and **(c) **prolyl 4-hydrolase-positive cells (Alexa 546, red), 15-LO-positive cells (Alexa 488, green), and superimposed stainings. Original magnification: (a, b) ×200 and (c) ×400.

We characterized the cellular distribution of the respective enzymes in RA synovial tissue. As shown by double immunofluorescence, 5-LO was mainly detected in synovial CD163^+ ^macrophages (Figure 3a) and in CD68^+ ^macrophages (data not shown), but not in fibroblasts. 5-LO expression was also detected in scattered CD66b^+ ^neutrophils and tryptase-positive mast cells (Figure 3b,c). 15-LO-1-positive staining was identified in macrophages, fibroblasts and CD31^+ ^endothelial cells (Figure 4). No staining was observed for either enzyme in CD3^+ ^T cells or in CD20^+ ^B cells (data not shown).

### The clinical response after intraarticular GC administration is associated with a decrease in 5-LO expression but not in 15-LO-1 expression in rheumatoid synovium

**Figure 5:**
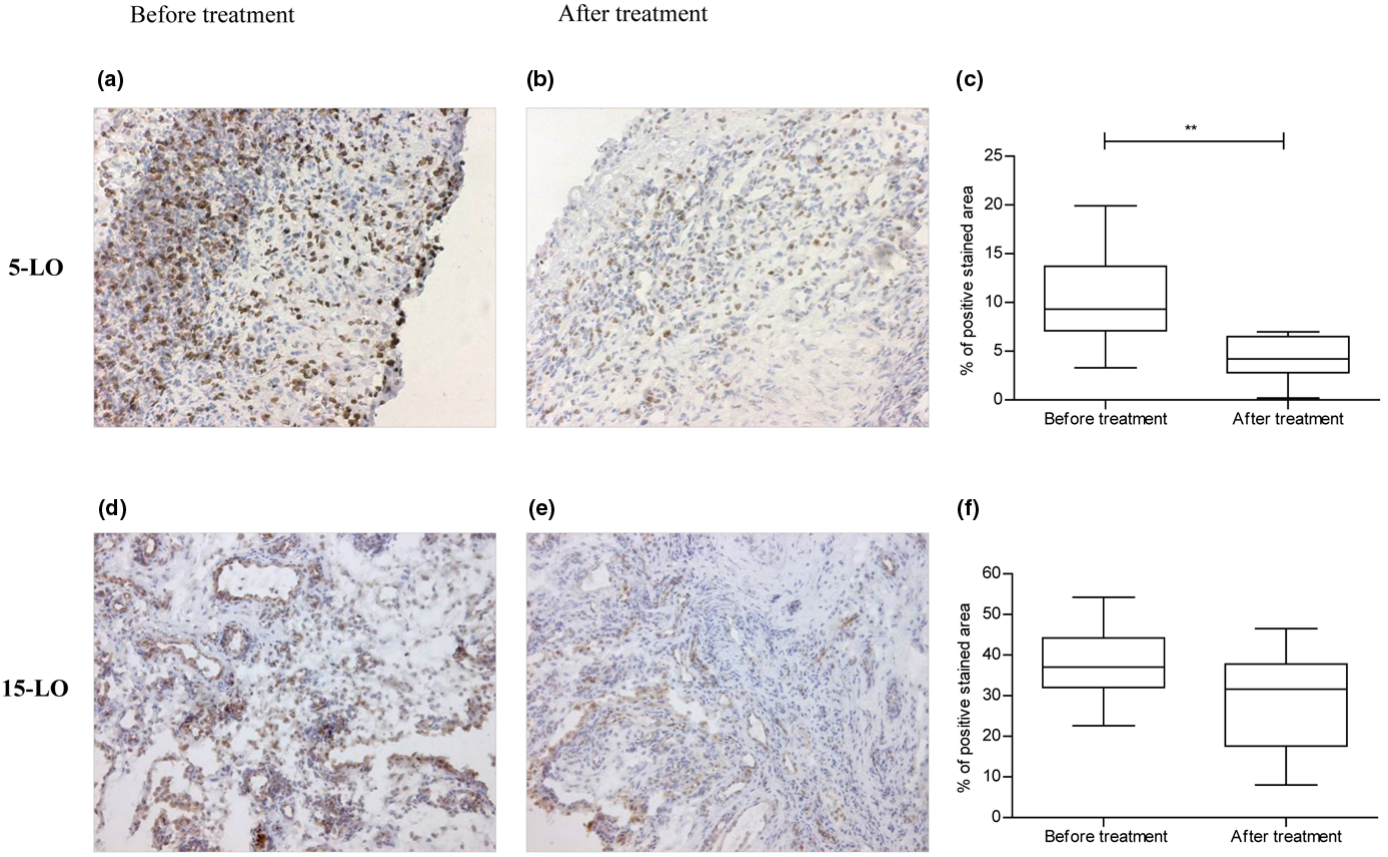
Intraarticular glucocorticoids effects on lipoxygenase expression in rheumatoid arthritis synovium. Intraarticular glucocorticoids decrease 5-lipoxygenase (5-LO) expression in rheumatoid arthritis (RA) synovium but leave unaltered the expression of 15-LO-1 enzyme. RA synovial tissue (n = 11) showing diaminobenzidine (brown) staining for 5-LO **(a) **before and **(b) **after treatment, and for 15-LO-1 **(d) **before and **(e) **after therapy (hematoxylin counterstained). Graphs show image analysis results for **(c) **5-LO and **(f) **15-LO-1 expression in synovial biopsy sections taken before and after intraarticular corticosteroid injection. Results expressed as percentage of the total area of counterstained tissue. Horizontal lines, median values; whiskers, range values. **P < 0.01. Original magnification: (a, b) ×125 and (c, d) ×160.

All patients included in the study were clinical responders as assessed by the arthroscopy-performing physician. Figure 5 demonstrates that intraarticular GCs significantly reduced the expression of 5-LO enzyme in the synovium (*P *= 0.002). By contrast, the 15-LO-1 enzyme displayed a reduced expression after therapy in nine out of 11 patients, while two patients had a higher expression. Overall in this analysis, however, the 15-LO-1 pattern did not significantly change following intraarticular corticosteroid therapy (Figure 5d to 5f).

### Synovial fluid cells express a functional 15-LO-1 enzyme and form 15-HETE

**Figure 6:**
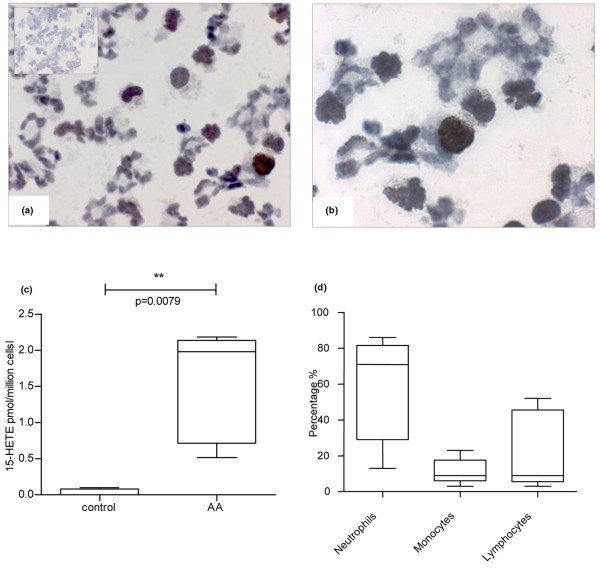
15-Lipoxygenase-1 expression in rheumatoid arthritis synovial fluid cells and 15-hydroxyeicosatetraenoic acid production. Rheumatoid arthritis (RA) synovial fluid cells express 15-lipoxygenase-1 (15-LO-1) and produce 15-hydroxyeicosatetraenoic acid (15-HETE) upon stimulation with arachidonic acid (AA). **(a, b) **Cytospin preparation of synovial fluid cells shows brown (diaminobenzidine) staining for 15-LO-1. Inset: isotype control. **(c) **15-HETE formation in control synovial fluid cells and synovial fluid cells incubated with AA. **(d) **Cellular composition of the RA synovial fluid showing the percentage of neutrophils, monocytes and lymphocytes in samples from five patients. Horizontal lines, median values; whiskers, range values. Original magnification: (a) ×500 and (b) ×800.

RA synovial fluid cells demonstrated strong positive staining for 15-LO-1 in mononuclear cells and possibly in neutrophils (Figure 6a,b). Direct measurement of the 15-HETE content in synovial fluid obtained from RA patients was not possible, however, since the concentrations were below the limits of detection (data not shown). We therefore analyzed the functional ability of 15-LO-1 in cells isolated from RA synovial fluid. Flow cytometry analysis has shown that synovial fluid cells are composed mainly of neutrophils (~70%), monocytes and lymphocytes. The cellular composition of the synovial fluid samples is shown in Figure 6d.

After incubation of synovial fluid cells with or without AA at 37°C for 5 minutes, 15-HETE could be measured (mean ± standard error of the mean (n = 5), 1.5 ± 0.03 pmol/10^6 ^cells compared with 0.08 ± 0.02 pmol/10^6 ^cells in controls) (Figure 6c). Any nonenzymatic 15-HETE present in AA or formed during the incubation period in corresponding buffer controls was subtracted from these results.

## Discussion

The leukotriene pathway, and in particular LTB_4_, has long been recognized to have deleterious effects in arthritis. Nevertheless, the enzymes responsible for arthritis formation have not been well characterized in synovial tissues, and nor is it known whether they are targeted by current RA therapy.

In the present study we showed that 5-LO is expressed in synovial tissue from patients with RA, mainly in macrophage-like cells and to a lesser extent in neutrophils and mast cells. We did not, however, detect 5-LO enzyme in T cells or B cells in RA biopsies. Although previous studies indicate that tonsillar B lymphocytes and B-cell lines are abundant in 5-LO protein [39,40], recent data reveal that, within the tonsils, it is the mantle-zone B cells that are 5-LO-positive and not the germinal-centre B cells or plasma cells [41]. In fact, it has been suggested that RA synovial B cells mainly represent mature activated memory B cells and plasma cells [42]. Our findings that RA CD20^+ ^B cells display no detectable 5-LO staining may therefore be explained in part by the specific B-cell subsets present in RA synovium. The wide expression of 5-LO in the synovial tissue of RA patients is in agreement with studies describing the LTB_4 _presence in RA synovial fluid [1] and the 5-LO-positive immunostaining in areas coinciding with macrophage localization [24].

We also observed a low number of cells expressing 5-LO in OA tissue, scattered in areas with more abundant synovial membranes. By quantifying the positive staining areas, we showed that OA synovium expresses significantly less 5-LO than RA tissue. Indeed, OA synovial fluid has been shown to contain less LTB_4 _than RA fluid [8] and OA synovium is known to contain a low degree of infiltrating inflammatory cells, which is in line with our observations.

There are a limited number of studies investigating the 15-LO-1 pathway in chronic inflammatory disorders, although the products of this pathway have long been recognized to play important roles in immune regulation and inflammation [43]. We underwent a detailed study characterizing the expression of 15-LO-1 enzyme in RA synovium, showing that it is highly expressed in synovial lining and scattered sublining fibroblast and macrophages and also in vessels of different sizes. In addition, we showed here that endothelial cells from both RA and OA biopsies express 15-LO-1. In OA, however, few synovial lining cells stained positively for 15-LO-1 while 15-LO-1 was abundantly present in vessels. The overall 15-LO expression was significantly lower in OA synovium compared with RA synovium.

The expression of functional 15-LO-1 in endothelial cells has been somewhat controversial, although some studies have demonstrated expression of 15-LO-1 in these cells [44]. Human and rabbit aortic endothelial cells, however, were more recently revealed to express 15-LO-1 mRNA and protein [45]. In addition, the presence of 15-LO-1 in endothelial cells was correlated with an induction of NF-κB activity and a subsequent increase in intracellular adhesion molecule expression [46], which may augment the local influx of cells. Our finding that 15-LO-1 is localized in endothelial cells from RA synovium may therefore be related to its ability to form mediators that locally attract immune cells and promote inflammation.

Although 15-LO-1 is largely present in the synovial tissue, its main product (15-HETE) was not detectable in synovial fluid in the present study. Synovial fluid cells incubated with AA form only small amounts of this eicosanoid product. One explanation for this may reside in the methodology we used, such as a short incubation time. Furthermore, the synovial fluid was isolated from patients treated with various regimens. Cells incubated with AA still form significantly higher amounts of 15-HETE compared with cells without AA, demonstrating the capacity of these cells to produce 15-HETE.

We further demonstrated that 5-LO expression in synovial tissue was significantly decreased following intraarticular administration of GCs. This finding is consistent with previous work documenting reduced synthesis of LTB_4 _in neutrophils of patients with RA after intraarticular GC injection [33]. It has been demonstrated previously that the number of macrophages in RA synovial tissue is not influenced by therapy with local GCs [47]. This suggests that the decrease in 5-LO expression we observe here most probably reflects a decrease in cellular expression and not a lower number of cells locally present. Other investigators, however, have found that systemic treatment with GCs is followed by reduced macrophage infiltration in RA synovium [48]. Different biological mechanisms may operate when administrating GCs intraarticularly or systemically. Further investigation is therefore needed to elucidate the mechanism for the reduction in 5-LO expression.

GCs are very efficient in achieving important clinical and radiographic outcomes in RA [49]. Intraarticular GC may also confer a bone-protecting effect in RA by decreasing the RANKL/osteoprotegerin ratio [50]. Previous studies have indicated LTB_4 _to be a negative regulator of bone metabolism by activating osteoclasts and inhibiting osteoblasts, thus promoting bone degradation and inhibiting bone formation [51,52]. In this context, the decrease in 5-LO expression after intraarticular GC therapy may indicate a potential role for 5-LO in bone degradation associated with inflammatory arthritis and suggests a new mechanism for the bone-protecting effects of intraarticular GCs.

Since LTB4 has been demonstrated to be a key regulator in the pathogenesis of murine arthritis [9], it may be conceivable that targeting the 5-LO pathway could provide additional benefits in the treatment of RA, by reducing the formation of LTB_4 _and, by this means, decreasing the chemotaxis of inflammatory cells. Few studies have investigated the effects of 5-LO pathway inhibition in RA patients. In a 4-week clinical trial, treatment with zileuton showed a trend towards clinical improvement, but the duration of the study was not adequate to assess efficacy [53]. Novel 5-LO inhibitors may possibly offer better treatment effects.

There are few studies to date on 15-LO-1 in RA, and the role of its products in inflammation is not clearly defined. We demonstrate here that locally administered corticosteroids do not significantly change the expression of 15-LO-1 in RA synovium. Previously, it was shown that 15-LO-1 metabolites confer proinflammatory actions by increasing vascular permeability *in vitro *[19], enhancing expression of monocyte chemotactic protein-1 and TNFα in vascular smooth muscle cells via activation of NF-κB [54]. On the other hand, 15-LO-1 products may also have anti-inflammatory properties, by reducing synovitis through decreased LTB_4 _in experimental arthritis [55], inhibiting chemotaxis of neutrophils to LTB_4 _[56] or through transcellular formation of lipoxins [57]. In this sense, it is noteworthy that IL-13, known to increase 15-LO-1 expression in several cell systems, is constantly present in synovial fluid of RA patients and has the ability to decrease proinflammatory cytokine production by synovial fluid mononuclear cells [58]. 15-LO-1 and its metabolites may therefore have dual roles in inflammation, and the net effect in RA needs further investigation.

## Conclusions

In the present study we have shown that RA synovium expresses 5-LO and 15-LO-1, and that administration of intraarticular corticosteroids is followed by a significant reduction in 5-LO expression while leaving the 15-LO-1 enzyme unaffected. Our results provide an additional explanation for the beneficial effects of local corticosteroids in RA, through inhibition of 5-LO enzyme and reduced formation of its proinflammatory products. Together with previous studies incriminating LTB_4 _as a potent mediator of joint inflammation and destruction in RA, the present study suggests the use of 5-LO inhibitors as add-on therapy.

## Abbreviations

15-HETE: 15-hydroxyeicosatetraenoic acid; AA: arachidonic acid; GC: glucocorticoid; IL: interleukin; LO: lipoxygenase; LTB_4_: leukotriene B_4_; OA: osteoarthritis; PBS: phosphate-buffered saline; RA: rheumatoid arthritis; RANKL: receptor activator of NF-κB ligand; TNF: tumor necrosis factor.

## Competing interests

The authors declare that they have no competing interests.

## Authors' contributions

KRG performed acquisition and interpretation of data, performed statistical analysis and wrote the manuscript. MK participated in acquisition and interpretation of data, and in writing the manuscript. AIC provided the patient biopsies and their clinical data and participated in writing the manuscript. LB participated in the collection of data. EaK provided patient biopsies and participated in writing the manuscript. H-EC participated in the study design and preparation of the manuscript. OR participated in writing the manuscript. P-JJ was responsible for study design, interpretation of data and participated in writing the manuscript.
